# The Integration of Technology into Treatment Programs to Aid in the Reduction of Chronic Pain

**Published:** 2016-12-31

**Authors:** Chad Eckard, Caitlyn Asbury, Brandon Bolduc, Chelsea Camerlengo, Julia Gotthardt, Lauren Healy, Laura Waialae, Ceirra Zeigler, Jennifer Childers, Joseph Horzempa

**Affiliations:** 1 Department of Graduate Health Sciences, West Liberty University, West Liberty, West Virginia, USA; 2 Department of Natural Science and Mathematics, West Liberty University, West Liberty, West Virginia, USA

**Keywords:** Pain management, Chronic pain, Opioids

## Abstract

In the United States, roughly $600 billion is spent on pain management – usually in the form of addictive opioid drugs. Due to the dangers associated with long-term opiate-based pain medication, the development of additional strategies for chronic pain management is warranted. The advent of smartphones and associated technology has provided healthcare providers with a unique opportunity to provide pain management support. This review summarizes of the use of technology to supplement chronic pain management regimens. Smartphone and internet-based applications that employ online journals facilitate improved communication between patient and clinician and allow for more personalized care and improved pain management. For instance, the e-Ouch application provides a platform for pain logs as well as feedback and coaching to patients via Twitter postings and blogs. Other applications provide online resources and blogs to improve patient education, which has shown to relieve patient symptoms through lifestyle modification. Internet-delivered cognitive behavioral therapy (CBT) focuses on the psychological coping mechanisms. The application of technology and smartphone apps toward pain management shows promise toward reducing the use of opioids in pain management, but has yet to be incorporated as a standard practice. More robust studies critically evaluating the efficacy of these technology-based therapies need to be conducted before standardization and insurance coverage can become reality.

## Introduction

In 2012, approximately 1 in 10 patients reported to experience chronic pain – having discomfort that persists for three consecutive months [1]. Although not considered to be a leading cause of morbidity or mortality in the United States, the treatment of chronic pain is among the most expensive [2]. For instance, the annual cost of pain treatment ($560 to $635 billion) is greater than the costs of heart disease ($309 billion), cancer ($243 billion), and diabetes ($188 billion) [2].

The prevalence of prescription opioid use is likely the major factor driving the cost of chronic pain in the United States. Twenty percent of patients reporting chronic pain not associated with cancer receive an opioid prescription [3]. Moreover, cancer patients utilize opioids at a rate of 30 to 50 percent for those undergoing active antineoplastic therapy, and 75 to 90 percent for those with advanced disease [4]. In 2012, healthcare providers wrote 259 million prescriptions for opioid pain medication [3]. Prescription opioid rates have quadrupled from 1999 to 2013 and this has led to an increase in deaths associated with the use of these drugs [3]. As studies indicated that overuse of these prescription drugs further exacerbates the utilization of illicit opiates, the Centers for Disease Control and Prevention concluded the level of prescription opioid use is fueling the current heroin epidemic in the United States [3]. Because of the dangers associated with chronic opiate-based pain medication, the development and implementation of alternative therapeutics and pain management strategies is warranted.

Clinicians treating patients experiencing chronic pain have access to multiple technology programs to supplement traditional therapy to improve the quality of life and improve the subjective pain scale of the patient [5]. In this review, we provide a summary of the use of technology in chronic pain management including pain diaries, various educational tools, programs to improve the cognitive behavioral aspect of pain, and programs using a combination of features. The integration of technology programs will aid in the reduction of chronic pain and perhaps the overuse of prescription opioids.

The primary objectives for increased use of “technology” in the healthcare setting are to improve patient outcomes, communication, and data availability for providers. Examples of recent technological additions to the healthcare community include: Electronic Medical Records (EMR), Patient Portals, Telemedicine, 3D Printing, Robotic surgery, Robotic Nurse Assistant, and Video-Game based Physical Therapy [6,7]. The time saved by using these advances can be applied toward improving patient care. Therefore, the implementation of this technology could alleviate chronic conditions which are straining healthcare costs and resources.

## Pain Diary

Recent developments in technology have given rise to the possibility of enhancing a patient's current chronic pain treatment plan using internet and smartphone applications (apps) to reduce pain [8]. The electronic pain diary smartphone apps such as WebMD Pain CoachTM and Habit Changer allow the patient to make journal entries three times each day – morning, midday and evening – using a scale to rate pain as well as log daily activities [9]. During follow-up appointments, the patient and provider review trend analyses produced by the application. The trends identified by the clinician will be used to alter the patient's current regimen and educate the patient on ways to improve their pain according to daily activities [9,10]. The benefit of using an electronic pain diary is the ability of this app to support an “n-of-1 trial” - a single-subject study where different treatment regimens are compared on the patient to attain the most effective therapy. The apps' ability to allow multiple n-of-1 trails enables a treatment plan tailoring the patient's unique case of chronic pain. Research has suggested that n-of-1 trials are the most beneficial methods in efforts to improve patient satisfaction of care and overall quality of life as well as cost effectiveness, yet this approach is not used regularly in the practice of medicine [11,12].

In addition to supporting n-of-1 trials, pain diaries also utilize realtime data entry which ensures better adherence to treatment regimens and produces more reliable data than the traditional approach of patient reporting from memory during appointments. The e-Ouch usability study was completed to examine the simplicity of use of pain diary apps for adolescents with arthritis [13]. In a comprehensive chronic pain management program, the e-Ouch app was designed to serve as a patient-provider bridge to most accurately report pain. Generally, pain scales only encompass intensity and not the multifaceted effect that chronic pain has on activities of daily living (ADL) [13]. Forty-four percent of chronic pain patients using the e-Ouch smartphone app reported an improvement in ADL. However, 34% reported unchanged pain, while 14.5% reported worse pain suggesting that although not universally effective, these apps do have potential to act as an aid in management of chronic pain for some patients [14].

Due to the daily variability of pain for each chronic pain patient, under- and over-treatment of pharmacological agents is a frequent possibility. The pain control sequence typically begins with administration of NSAIDS, and escalates to use of opioids when the analgesia diminishes or is ineffective. Increasing doses of opioids are often administered as pain returns. In an effort to use technology and n-of-1 trials to better monitor the variable course of each patient's pain treatments, the PREEMPT study utilized the “Trialist” pain diary app to allow patients to take an active part in their healthcare. Based on feedback from this smartphone app, the provider-patient team may analyze pain patterns and choose the most effective treatment between two potential treatment plans. The pain diary app results are used in an n-of-1 fashion, allowing the provider and patient to review a statistical analysis of the trial results and determine the best care plan. As many pain management apps are currently being modified, studied, and improved for clinical success, the use of smartphones in pain management creates great potential for individualized care based on personal empirical data [10].

## Educational Tools

Internet-based patient education supplements traditional chronic pain treatment strategies by emphasizing self-management. The goal of such therapy is to create a form of independent learning for the patient to allow them to comprehend their pain and what exacerbates their symptoms. These programs, such as twitter blogs, smartphone applications, and other online resources, can be individually tailored to the patient and be accessed for little to no cost. Sources of information can be in the form of social media and/or blog style websites, such as in a 2016 observational study [15] of chronic pain patients. This study included 200 patients and evaluated the use of internet blogs, twitter postings, and other online educational resources as self-management educational tools in conjunction with their current pain medication. Patients were required to read about safe medication use, dosing, supplement use, eating habits, and managing stress for 15 minutes a day. Moderate improvements were seen in the patients' pain, anxiety, depression, and quality of life compared to baseline after 6 months. Forty-five percent of patients reported a reduction of moderate to severe pain following treatment using the educational tools (P<0.0001). Fifty percent of patients reported a decrease in depression (P<0.0001) and 30% reported a reduction in anxiety (P<0.0001). Thirty-five percent of patients reported an improvement in their overall quality of life following treatment (P<0.0001). This study suggests that self-management educational tools can improve outcomes of patients afflicted with chronic pain. Although these results seemed promising, this study did not implement a control group. Lack of these comparisons warrants further research with a larger population and control groups. Nevertheless, internet-based educational tools can allow patients to take a self-paced approach to learning about chronic pain and how to manage this disease most efficiently in conjunction with their current pain medication [15].

Additional online tools including the Pain Course, an internet-derived pain management program, provides online education lessons to help patients understand their symptoms and difficulties. The lessons along with homework assignments teach self-management skills applicable to the patient's condition for management of flares, reducing pain, and anxiety [16]. Patient-centered complementary techniques that involve the patient and offer the patients a proactive role have the potential to provide more efficient and thorough pain management. Incorporating the patient into the treatment strategy as well as increasing their knowledge on the management of their chronic pain will help to reduce the workload experienced by the clinician. Patients will be able to spend more time away from the provider's office working on the management of their chronic pain. This will, in turn, decrease the workload on the provider by decreasing the amount of office visits and time spent with the patients pertaining to their pain treatment.

## Cognitive Behavioral Therapy

Internet-delivered cognitive behavioral therapy (CBT) programs show promise in helping patients cope with their pain and other associated sequelae such as depression, anxiety, and stress [16]. In-person therapy is not always an option for patients dealing with chronic pain due to geography or other practical reasons. Internet-based CBT provides a comparable alternative, delivered to a patient through his or her mobile device. CBT focuses on identifying patients' perceptions about their pain then altering those ideas to further influence behavior [17]. Depression, stress, and anxiety in chronic pain patients adversely affect the patients' quality of life and further exacerbates their symptoms. By learning to alter their ideas about pain and behave differently, the patients' frequency of episodes and the other associated sequelae may decrease.

Clinician support is also vital in this line of therapy [16]. In one study conducting internet-based CBT, clinician contact was mediated through email or chat rooms. Patients in all contact groups reported improvements in their degree of disability, depression, anxiety, and average pain [16]. With this therapy being conducted on the patient's own time and schedule, clinician contact with the patient is crucial in case of any questions or concerns regarding the program arise. The needs of each patient will differ, so individualized treatment plans would address the needs of the patient and the clinician contact time would be considered as well.

Trials that evaluated pediatric patients revealed that CBT helped this population to manage pain, indicating that these technology-based programs can be effective across multiple demographics. In this age group, replacing the thought, feeling, and action of the pain with internet-based therapy helped reduced the symptoms and change cognitive thoughts to be more positive toward the perception of pain. Using this method in conjunction with self-management techniques, like deep breathing and compliance to the program, help to offset the negative aspects of pain proved to be a reliable appropriate method. However, more research is needed to determine the interrelationship between adolescent motivation/readiness to change and participation with internet-based interventions.

External support has also been used for online based pain management CBT. Support systems such as an online coach (someone whom the child can message and talk to about their pain everyday) have been used in behavioral interventions for youth with chronic illness. For instance, a greater number of messages sent by adolescents demonstrating rapport or therapeutic content predicted positive treatment outcomes compared to those who did not. Therefore, interaction with an online coach may increase the benefit of this internet-based behavioral pain management treatment program for adolescents in a chronic pain management situation [18].

The use of this self-management approach is associated with adherence to the CBT replacement treatment guidelines [18]. A provision that supported compliance required patients to message their online coach once a week and to log onto the online program daily. Taking time out each day to report a pain score to their online coach provided a sense of rapport and responsibility for self-management in treating the chronic pain. In doing so, this supported open communication about what may be triggering pain episodes. Patients who completed the online commitments exhibited an association with the amelioration of chronic pain. Because the external support system and personal responsibility to manage the pain daily often support one another, this approach shows real promise for the future of pain management [18].

## Combined Approach

Some treatment regimens use technology to provide education, support, documentation, and feedback, to supplement traditional medical therapies. By taking an active role in their pain management while medicating, patients are empowered with a greater sense of control [15]. Utilizing various internet-based self-management activities creates more opportunities for patients to confront and overcome their pain. The internet-based activities also generate an avenue for individuals with little access to specialized facilities [15].

An observational study of 200 patients who had reached a plateau in chronic pain management for at least 12 months evaluated outcomes after incorporating technology applications into their current treatment. Patients were encouraged to access an educational blog and twitter postings under the hashtag “zendose” to practice safe activity, positive thinking, improved eating habits, manage stress, learn mindfulness skills, and review proper medication and supplement use [15]. Patients received email and phone reminders to improve compliance with scheduled appointments and they kept logs to record pain and anxiety levels. Logs were reviewed by a clinician and used in discussion during appointments. Patients who used the program incorporating the applications reported a higher quality of life with a significant decrease in pain, depression, and only moderate anxiety compared to their recorded scores at the beginning of the study [15].

In another study in which patients used technology to supplement pain medication, subjects participated in a smartphone-based intervention consisting of 1 face-to-face session followed by 4 weeks of written communication [19]. Participants generated 3 smartphone diary entries daily to promote their awareness of and to reflect on pain-related thoughts, feelings, and activities. The registered diaries were immediately available to a therapist who submitted personalized written feedback daily based on cognitive behavioral principles. Both groups were given access to a non-interactive website after discharge to promote constructive self-management. Outcomes were measured with self-reported questionnaires. Compared to the control group, participants reported a lower incidence of “catastrophizing” events, greater pain “acceptance”, improved functionality, and reduced symptom levels further supporting that supplementation of pain medication with technology is effective [19].

## Discussion

The addition of technological therapies into a patient's existing treatment regimen creates both drawbacks and benefits for the patient and clinician [20]. An improved line of communication between providers and patients will advance the management of chronic pain [18,21]. This allows patients to better understand and self-manage their pain as well as improve psychological aspects such as depression and anxiety. Implementation of technology allows providers to utilize more innovative approaches for managing their patients' pain. Trend analysis of the data will allow the provider to get a more detailed view of the patient and their symptoms to most effectively treat the pain [10,12]. Pain diary entries of all the patients will help document the patient's severity and frequency of symptoms [13,14,22]. Utilization of the aforementioned technology will allow more efficient communication between the provider and patients pertaining to changes in treatment regimens and less time for providers to document and analyze pain trends [23].

Incorporation of internet and smart-phone based tools will make pain management more accessible to the patient [24]. These tools may be utilized from home or anywhere else with internet service. Many patients work full-time jobs and are faced with a decision to either work or seek care for their chronic pain. Lack of transportation and long travel distances can present as obstacles for receiving care. These restrictions can be avoided by the accessibility of internet and smart-phone based intervention by allowing patients to conduct these treatments at their own convenience from virtually anywhere [25]. Utilizing technology increases the availability of additional treatment services to which the patients may not have access. Alternative therapeutic methods, such as physical therapy, may create an additional out of pocket expense for the patient. The higher out of pocket cost of such alternative therapies could drive more people to seek prescription pain medication covered by their insurance. Despite these drawbacks, internet-based programs used in conjunction with pain medication can improve patient pain outcomes, ultimately lowering the reliance on opioids [15].

While internet-based treatment methods reduce discomfort and anxiety of chronic pain patients, there are disadvantages as well. A main concern for this method is the unknown source of advice for online webpages and apps [26]. Based on the lack of designer credentials, patients may be receiving input from an untrustworthy source which may negatively impact their progress [9]. Another disadvantage is the lack of a standardized pain management scales, such as Visual Analogue scale, Face Rating Scale, and Fibromyalgia Impact Questionnaire. By not standardizing the patient's pain, treatment outcomes cannot be successfully quantified. Furthermore, additional, more robust studies are necessary to fully vet the validity of the utilization of specific apps for the treatment of chronic pain [27]. Technological barriers in the geriatric [28] and pediatric population [29] are a significant concern for practitioners, but research suggests that apps are “user friendly” when an appropriate age-targeted app is chosen [15]. Ultimately, all patients will need an orientation for the new application, which initially increases patient-provider contact time for a short period until use of the app has been mastered [9,15]. This could possibly delay pain management and produce an initial burden on clinicians.

## Conclusion

Adding an internet application featuring a pain diary, educational readings, cognitive behavioral activities or a combined therapy to a patient's existing chronic regimen can be beneficial in helping reduce patient symptoms. Furthermore, scientific evidence is not strong enough to suggest use the apps without other treatment regimens in reducing symptoms, and there is a need for more research to discover the full potential the apps may have for pain management in the medical field. At this current time, there is a lack of research on the apps that includes large study populations. However, investigations are currently being conducted, such as the e-Ouch trial, that will provide more robust data for health care providers [13]. Additionally, many software platforms, including Apple and Android, offer many downloadable pain management applications for cell phones as well as personal computers that can be utilized by clinicians, but many apps are not regulated or designed by medical professionals [9]. To integrate the application into clinical practice, future applications should be created with the expert opinions of pain management health care providers to ensure the application meets medical standards. Therefore, clinicians should be very cautious when choosing apps for patient use and need to actively research which application has the most evidence-based research proving the reliability. Evaluations of the reliability and usability of many applications have been published, so health care providers can make an educated decision for their patients [8,27]. The idea of utilizing smartphone applications in chronic pain management is both innovative, appears to be effective, and holds greater potential in chronic pain management with awareness and proper integration.
